# Comparison of Conventional Total Knee Arthroplasty Versus Robotic-Assisted Total Knee Arthroplasty in Primary Osteoarthritis of the Knee: A Prospective Comparative Pilot Study

**DOI:** 10.7759/cureus.117259

**Published:** 2026-09-30

**Authors:** Chirag Kapoor, Malkesh Shah, Kunj R Patel, Devanshu Garg, Tarun Das, Sankalp S Jain, Tanmay A Bhagwatkar

**Affiliations:** 1 Department of Orthopaedics, Dhiraj Hospital, Smt. B. K. Shah Medical Institute &amp; Research Centre, Sumandeep Vidyapeeth (Deemed to be University), Vadodara, IND

**Keywords:** conventional total knee arthroplasty, indian population, kinematic alignment, knee society score, primary osteoarthritis, robotic-assisted total knee arthroplasty

## Abstract

Background: Total knee arthroplasty (TKA) is a highly effective intervention for advanced primary knee osteoarthritis. Conventional TKA (C-TKA) has been the standard of care for decades, while robotic-assisted TKA (RA-TKA) aims to enhance surgical precision and alignment. Prospective Indian data comparing the two are scarce. We therefore conducted a small pilot comparison intended to generate preliminary estimates and inform the design of a future adequately powered randomised controlled trial, rather than to guide current clinical practice.

Aim: To obtain preliminary comparative estimates for conventional and robotic-assisted TKA in primary knee osteoarthritis with respect to intraoperative parameters, postoperative pain, functional outcomes, range of motion and complications in an Indian tertiary-care setting, as preparatory work for a properly powered randomised trial.

Materials and Methods: A prospective, non-randomised comparative pilot study was conducted on 30 patients with primary knee osteoarthritis (Kellgren-Lawrence grade 3 or 4). Fifteen patients underwent C-TKA with mechanical alignment, and 15 underwent RA-TKA with kinematic alignment; allocation followed patient choice after counselling on both techniques and their costs, and no formal sample-size calculation was performed. Outcomes assessed were tourniquet time, postoperative drain collection, Visual Analogue Scale (VAS) pain score, Knee Society Score (KSS) and knee range of motion in the immediate postoperative period and at 2 weeks and 1, 2 and 3 months. Analysis was performed in IBM SPSS version 26.0, with the Bonferroni method applied to the five repeated time-point comparisons (adjusted significance threshold p < 0.010).

Results: Knee Society Scores were significantly higher in the RA-TKA group than in the C-TKA group at every early follow-up time point, and all of these differences remained significant after Bonferroni correction. The between-group gap was widest at 2 weeks (76.9 ± 3.3 versus 87.9 ± 3.5; t = 8.86, df = 28, p < 0.001) and had narrowed by 1 month (97.2 ± 1.7 versus 98.7 ± 1.2; t = 2.79, df = 28, p = 0.006); by 3 months both groups reached a mean KSS of 100. VAS pain scores followed the same pattern and reached 0 in both groups by 3 months. Complications comprised one superficial surgical-site infection (n = 1/15; 6.7%) and three cases of prolonged pain (n = 3/15; 20.0%) in the C-TKA group and one pin-tract infection (n = 1/15; 6.7%) in the RA-TKA group; all were minor and had resolved by final follow-up, and the difference between groups was not statistically significant (Fisher exact test, two-tailed p = 0.330) in a sample of this size.

Conclusion: In this pilot cohort, the RA-TKA group had lower drain collection, lower early pain scores, and higher early Knee Society Scores than the C-TKA group despite a longer tourniquet time, with convergence by 3 months. No clinical recommendation follows from these observations. The sample was small and non-randomised; the two groups differed in alignment philosophy as well as in surgical platform, no radiographic alignment data were obtained, and no cost figures were collected, so the economic comparison remains entirely unquantified. The value of this study lies in the preliminary effect estimates it provides for sample-size calculation in an adequately powered randomised controlled trial with radiographic endpoints, formal health-economic evaluation, and follow-up of at least one year.

## Introduction

Knee osteoarthritis (OA) is a leading cause of musculoskeletal disability and a substantial burden on healthcare systems [1]. Total knee arthroplasty (TKA) is a highly effective intervention for end-stage knee OA, relieving pain and restoring function [2]. Demand is rising globally [3], and India shows a similar trend driven by an ageing population and lifestyle changes [4].

Conventional, or manual, total knee arthroplasty (C-TKA) uses mechanical alignment guides and jigs for bone cuts and component positioning and has long been the gold standard, with excellent long-term survivorship [5]. However, achieving consistent alignment is challenging, and malalignment is linked to abnormal wear, component loosening, persistent pain and revision [6,7]. Soft-tissue balancing also depends heavily on the surgeon's experience and subjective feel [8].

Robotic-assisted total knee arthroplasty (RA-TKA) was developed to address these limitations [9]. Using patient-specific preoperative planning and intraoperative robotic guidance, it aims to improve the accuracy of bone preparation, component positioning and soft-tissue balancing [10]. Available platforms are active, semi-active (haptic) or passive systems [11]. The goal is more accurate, reproducible implant placement and consistent limb alignment, potentially improving outcomes, satisfaction and implant longevity [12].

TKA volumes are rising sharply in India [13], where patients often present with severe deformities and culturally important demands such as squatting and cross-legged sitting that require precise surgical execution [14]. RA-TKA adoption in India remains limited, mainly because of the high cost of imported systems and the need for specialised training [15]. Comparative Indian data are therefore needed to inform practice and policy [16].

The present pilot study compared C-TKA and RA-TKA in patients with primary osteoarthritis of the knee with respect to tourniquet time, postoperative drain collection, visual analogue scale pain score, Knee Society Score and knee range of motion in the immediate postoperative period and at two weeks and one, two and three months, together with complications, in an Indian tertiary-care setting. It was conceived as preparatory work rather than as a definitive comparison: the sample was small, allocation was not randomised, and the two groups also differed in alignment philosophy, mechanical in the C-TKA group and kinematic in the RA-TKA group, which is treated throughout as a co-intervention. No formal costing was undertaken, so cost is discussed only as context for access. The intention is to provide preliminary effect estimates from which an adequately powered randomised controlled trial in the Indian population can be designed.

## Materials and methods

Ethics and study design

This was a prospective, non-randomised comparative pilot study conducted from July 2025 to April 2026 in the Department of Orthopaedics, Dhiraj Hospital, Smt. B. K. Shah Medical Institute and Research Centre, Vadodara, a single tertiary-care centre in India. It was designed as exploratory preparatory work for a future randomised controlled trial; no formal sample-size calculation was performed, and the cohort of 30 patients reflects the number treated by the participating surgeons within the study window rather than a powered target. Institutional Ethics Committee approval was obtained prior to the commencement of the study, and written informed consent was obtained from all participants. Thirty patients with primary osteoarthritis of the knee meeting the eligibility criteria were enrolled and assigned to one of two surgical interventions, conventional total knee arthroplasty (C-TKA, n = 15) or robotic-assisted total knee arthroplasty (RA-TKA, n = 15), after detailed preoperative counselling regarding both techniques and their costs. Allocation was not randomised, and no allocation-concealment procedure was used: after detailed preoperative counselling on both techniques and their respective costs, each patient selected the procedure they wished to undergo, so group assignment reflected patient preference and affordability.

Inclusion criteria

Patients diagnosed with primary osteoarthritis of the knee of Kellgren and Lawrence radiographic grade 3 or grade 4, who had failed at least six months of conservative treatment (analgesics, intra-articular injections, physiotherapy and lifestyle modification), were included. All included patients were medically fit for elective major lower-limb arthroplasty on routine pre-anaesthetic evaluation and aged between 50 and 80 years.

Exclusion criteria

Patients who had undergone conversion arthroplasty or a unicompartmental knee arthroplasty were excluded. Patients with inflammatory arthropathy such as rheumatoid arthritis or any other autoimmune joint disease, post-traumatic osteoarthritis or TKA performed for fracture, a previous infection of the affected knee joint, an underlying active knee joint infection, or a history of previous knee surgery (including high tibial osteotomy or any prior reconstructive procedure on the same knee) were not enrolled. Patients with osteoarthritis of Kellgren and Lawrence grade 1 or 2 were also excluded since elective TKA is not indicated at that severity. Patients with uncontrolled medical comorbidities, those unfit for general or regional anaesthesia, and those unable or unwilling to attend the scheduled follow-up visits were also excluded.

Surgical procedure

All procedures were performed under combined spinal-epidural anaesthesia by senior orthopaedic surgeons experienced in both techniques. A standard medial parapatellar approach was used. In the C-TKA group, mechanical alignment was achieved using intramedullary femoral and extramedullary tibial alignment guides to make the bone cuts (Figure 1). In the RA-TKA group, a pre-operative CT scan was obtained, and preoperative planning and intraoperative guidance were performed on the basis of the patient-specific 3D CT-guided model; the bone cuts were performed using the robotic system after registration with the patient's intraoperative anatomy through tibial and femoral tracking pins (Figure 2). In the RA-TKA group, a kinematic alignment (KA) strategy was employed, wherein implant components were positioned to replicate the patient's native joint anatomy and restore the pre-arthritic joint line, rather than imposing a fixed neutral mechanical axis as in the C-TKA group [17]. The same posterior-stabilised, cemented total knee implant system was used in both groups, with patellar resurfacing reserved for grade III or IV chondral changes. A standard suction drain was inserted and a layered closure performed in both groups. Postoperative analgesia, antibiotic prophylaxis, deep-vein-thrombosis prophylaxis and physiotherapy followed an identical protocol for both groups. The two groups therefore differed simultaneously in two respects, the instrumentation platform and the alignment philosophy, and this co-intervention was a fixed feature of the study design rather than an incidental observation.

**Figure 1:**
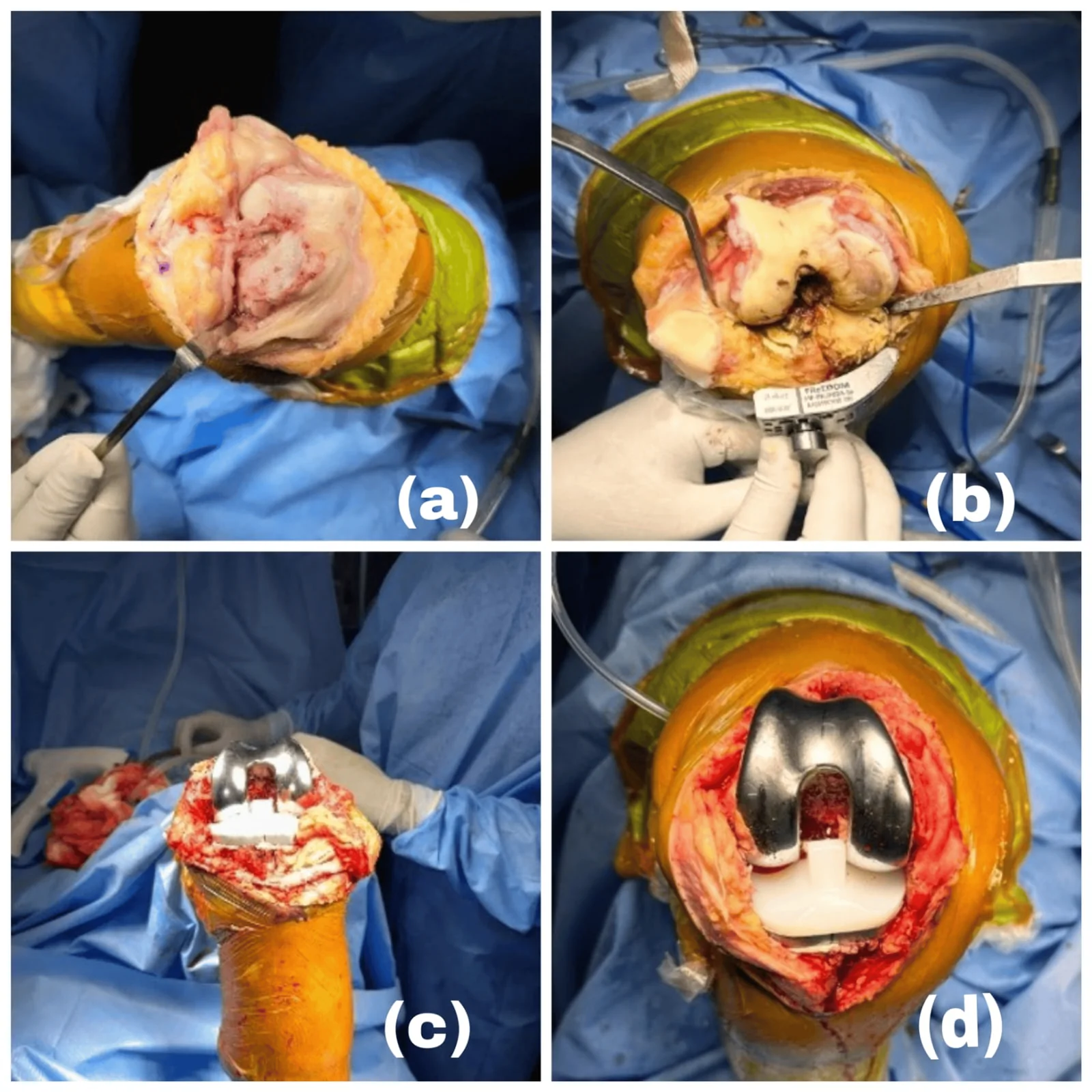
Intraoperative photographs of conventional total knee arthroplasty (C-TKA). Panels (a) and (b) show the anatomical exposure of the knee joint after capsulotomy and dissection through a standard medial parapatellar approach, with intramedullary and extramedullary alignment guides being applied for bone cuts. Panels (c) and (d) show implant insertion: trial femoral and tibial components are visualised in panel (c) and the final cemented posterior-stabilised tibial and femoral components with the polyethylene insert are visualised in panel (d).

**Figure 2:**
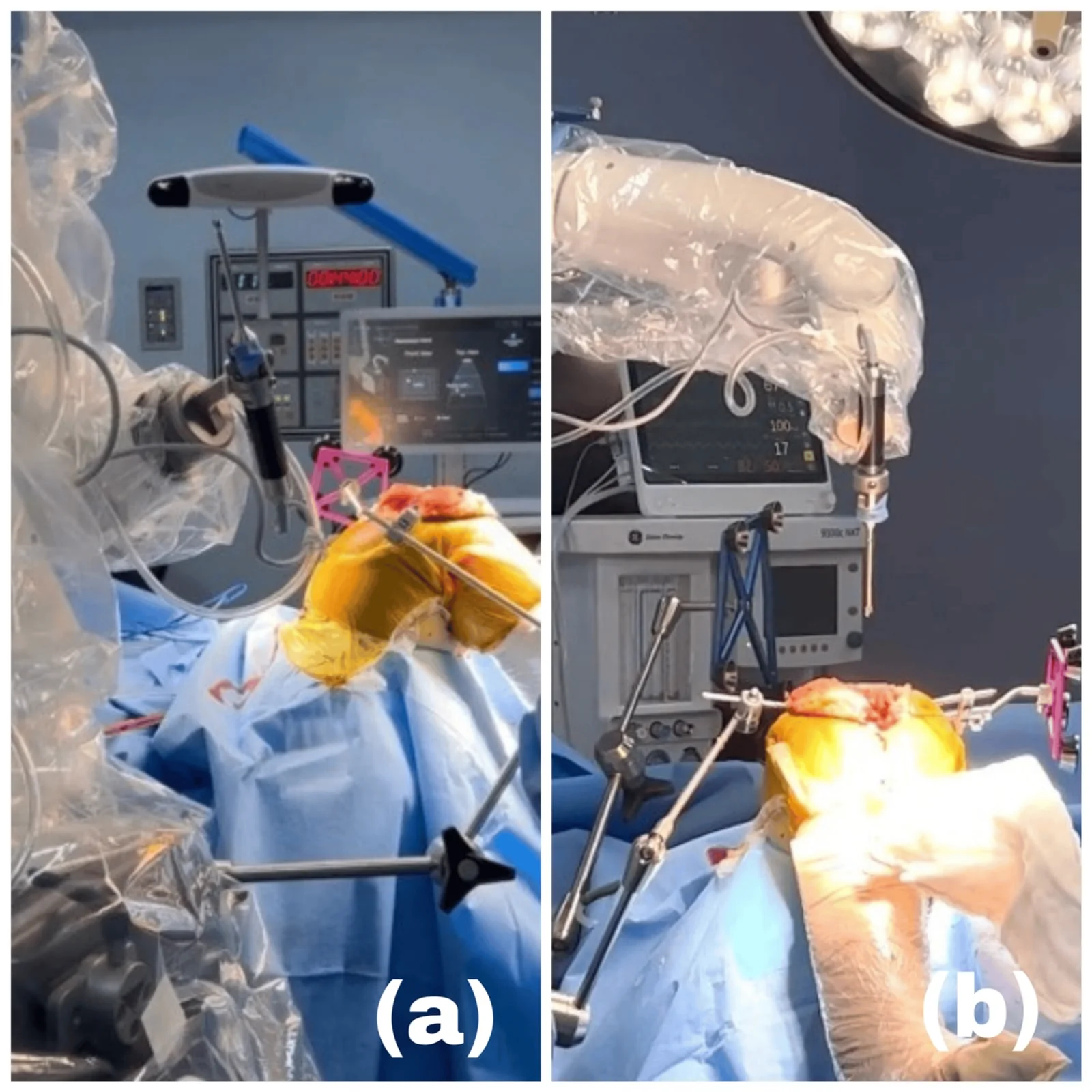
Intraoperative photographs of robotic-assisted total knee arthroplasty (RA-TKA). Panel (a) shows the robotic operative set-up with the optical tracking system and intraoperative planning console used for real-time intra-articular registration. Panel (b) shows the robotic arm executing the planned bone cuts under surgeon control, with tibial and femoral tracking pins clearly visible.

Outcome measures and scoring systems

Pre-operative radiographic severity of osteoarthritis was graded using the Kellgren and Lawrence classification [18]. Postoperative pain was assessed in the immediate postoperative period and at 2 weeks, 1 month, 2 months and 3 months using the Visual Analogue Scale (VAS) [19], where 0 indicates no pain, and 10 indicates the worst imaginable pain. Functional outcomes were assessed using the original Knee Society Clinical Rating System (Knee Society Score, KSS) exactly as described in the original publication by Insall et al. [20], in the immediate postoperative period and at the same follow-up time points. All three assessment instruments used in this study, namely the Kellgren and Lawrence radiographic grading system [18], the Visual Analogue Scale for pain [19] and the original 1989 Knee Society Clinical Rating System [20], were applied in their originally published form without any modification and are cited to their original source publications.

Tourniquet time was recorded from tourniquet inflation to deflation. Intraoperative blood loss was eliminated by tourniquet use; all blood loss measurements therefore represent postoperative drain collection volume. Any complications (superficial or deep surgical-site infection, deep vein thrombosis, neurovascular injury, periprosthetic fracture, thromboembolic events, prolonged pain, or pin-tract infection) were recorded. Postoperative radiographic alignment was not assessed: no postoperative measurement of the hip-knee-ankle axis or of coronal and sagittal component orientation was made, and the study therefore contains no direct data on surgical accuracy. No health-economic evaluation was undertaken either; itemised inpatient billing, implant and consumable costs, the additional imaging and disposables specific to the robotic workflow, and indirect costs such as time to return to work were not collected, and no cost figures are reported.

Statistical analysis

Demographic and clinical data were summarised using descriptive statistics. Continuous variables are expressed as mean and standard deviation (SD) together with the range, and categorical variables as absolute counts with the corresponding percentage, n (%). The normality of continuous data was assessed using the Shapiro-Wilk test; all continuous variables satisfied the assumption of normality, and between-group comparisons of continuous variables (tourniquet time, postoperative drain collection, Knee Society Score, visual analogue scale score and knee range of motion at each time point) were therefore performed throughout using the independent-samples Student's t-test. For every continuous variable, the value of the test statistic (t) and its degrees of freedom are reported alongside the corresponding p-value in the text and in the table legends.

Categorical variables, including complication rates, were compared using the Fisher exact test, applied in preference to the Pearson chi-square test because expected cell counts were less than five in all categorical comparisons; the Fisher exact test yields an exact two-tailed probability and does not generate a test statistic; therefore, only the p-value is reported for those comparisons. A two-tailed p-value of less than 0.05 was considered statistically significant for the single-occasion comparisons of tourniquet time, drain collection and complication rates. Pain, Knee Society Score, and range of motion were each compared between groups at five sequential time points, so the Bonferroni method was applied to control the family-wise error rate across those repeated comparisons, giving an adjusted significance threshold of p < 0.010 (0.05/5). Unadjusted p-values are reported throughout in the text and tables, and significance for the repeated comparisons is declared against the adjusted threshold.

Statistical analysis was carried out using IBM SPSS Statistics for Windows, version 26.0 (IBM Corp., Armonk, NY), and data were tabulated in Microsoft Excel (Microsoft Corp., Redmond, WA).

## Results

A total of 30 patients with primary osteoarthritis of the knee (Kellgren-Lawrence grade 3 or 4) underwent primary total knee arthroplasty during the study period. Fifteen patients (group C-TKA) underwent conventional total knee arthroplasty and 15 patients (group RA-TKA) underwent robotic-assisted total knee arthroplasty. No patient in either group was lost to follow-up, and complete five-time-point data were available for all 30 enrolled participants. Demographic characteristics, intraoperative parameters, functional outcomes, range of motion and complications are summarised in the tables below.

The mean age was 64.80 ± 6.53 years in the C-TKA group and 67.73 ± 6.66 years in the RA-TKA group. The sex distribution was 9 men (60.0%) and 6 women (40.0%) in the C-TKA arm and 7 men (46.7%) and 8 women (53.3%) in the RA-TKA arm. Mean body mass index was 27.98 ± 3.08 kg/m² in the C-TKA arm and 29.63 ± 3.05 kg/m² in the RA-TKA arm. The two groups were comparable on age, sex and BMI. The summarised demographic data are presented in Table 1.

**Table 1:** Demographic characteristics Age bands are mutually exclusive (50-60 = 50-59 yrs; 60-70 = 60-69 yrs; 70-80 = 70-79 yrs). All patients fell within the 50-80-year inclusion range. M, male; F, female; BMI, body mass index; SD, standard deviation.

Parameter	C-TKA (n=15)	RA-TKA (n=15)
Age group (years), n (%)		
50-60	4 (26.7%)	2 (13.3%)
60-70	7 (46.7%)	7 (46.7%)
70-80	4 (26.7%)	6 (40.0%)
Mean age ± SD (years)	64.80 ± 6.53	67.73 ± 6.66
Age range (years)	54–75	58–78
Sex, n (%), M / F	9 (60.0%) / 6 (40.0%)	7 (46.7%) / 8 (53.3%)
Mean BMI ± SD (kg/m²)	27.98 ± 3.08	29.63 ± 3.05

Mean tourniquet time was 84.7 minutes (range 80-88) in the RA-TKA group compared with 67.6 minutes (range 60-75) in the C-TKA group, a statistically significant difference of 17.1 minutes (independent-samples Student's t-test: t = 13.53, df = 28, p < 0.001). No intraoperative blood loss occurred in either group due to tourniquet application; the postoperative drain collection averaged 287.7 mL in the C-TKA arm and 255.0 mL in the RA-TKA arm (independent-samples Student's t-test: t = 8.13, df = 28, p < 0.001). Intraoperative parameters are summarised in Table 2.

**Table 2:** Intraoperative parameters in the C-TKA and RA-TKA groups. Data are mean (range). Between-group comparisons were performed using the independent-samples Student's t-test (df = 28); the value of the t statistic is reported alongside each p-value. p < 0.05 was considered statistically significant. C-TKA, conventional total knee arthroplasty; RA-TKA, robotic-assisted total knee arthroplasty.

Parameter	C-TKA (n=15)	RA-TKA (n=15)	Difference	Test statistic	p-value
Mean tourniquet time (minutes)	67.6 (range 60-75)	84.7 (range 80-88)	RA-TKA 17.1 min longer	t = 13.53 (df = 28)	<0.001
Intraoperative blood loss	None (tourniquet applied)	None (tourniquet applied)	Not applicable	Not applicable	Not applicable
Postoperative drain collection (mL)	287.7 (range 265-310)	255.0 (range 240-270)	RA-TKA 32.7 mL less	t = 8.13 (df = 28)	<0.001

Knee Society Scores were significantly higher in the RA-TKA group than in the C-TKA group at every early follow-up time point and converged to a mean of 100 in both groups by 3 months. Mean KSS values were 55.2 versus 60.5 (t = 3.54, df = 28, p < 0.001) in the immediate postoperative period, 76.9 versus 87.9 (t = 8.86, df = 28, p < 0.001) at 2 weeks, 97.2 versus 98.7 (t = 2.79, df = 28, p = 0.006) at 1 month, and 98.6 versus 99.9 (t = 4.42, df = 28, p = 0.001) at 2 months for C-TKA versus RA-TKA, respectively; all four comparisons were performed using the independent-samples Student's t-test. VAS pain scores were significantly lower in the RA-TKA group at every follow-up time point through two months and converged to 0 in both groups by three months. All of these between-group differences remained significant after Bonferroni correction for the five time-point comparisons (adjusted threshold p < 0.010). Functional outcomes are summarised in Table 3.

**Table 3:** Functional outcomes (Knee Society Score and VAS pain score) at all follow-up time points. Values are mean ± standard deviation. Between-group comparisons were performed using the independent-samples Student's t-test (df = 28); the value of the t-statistic is reported alongside each p-value. Because each outcome was compared at five sequential time points, significance is declared against a Bonferroni-adjusted threshold of p < 0.010; unadjusted p-values are shown. ‘Not applicable’ indicates no test was performed because values were identical in both groups. SD, standard deviation; VAS, visual analogue scale; C-TKA, conventional total knee arthroplasty; RA-TKA, robotic-assisted total knee arthroplasty.

Outcome Measure	Time Point	C-TKA (mean ± SD)	RA-TKA (mean ± SD)	Test statistic	p-value
Knee Society Score	Immediate post-op	55.2 ± 4.0	60.5 ± 4.2	t = 3.54 (df = 28)	<0.001
	2 weeks	76.9 ± 3.3	87.9 ± 3.5	t = 8.86 (df = 28)	<0.001
	1 month	97.2 ± 1.7	98.7 ± 1.2	t = 2.79 (df = 28)	0.006
	2 months	98.6 ± 1.1	99.9 ± 0.3	t = 4.42 (df = 28)	0.001
	3 months	100.0 ± 0.0	100.0 ± 0.0	Not applicable	Not applicable
VAS Pain Score	Immediate post-op	6.5 ± 0.8	5.4 ± 0.8	t = 3.77 (df = 28)	0.002
	2 weeks	4.3 ± 0.7	2.2 ± 0.6	t = 8.82 (df = 28)	<0.001
	1 month	2.1 ± 0.6	0.9 ± 0.3	t = 6.93 (df = 28)	<0.001
	2 months	0.9 ± 0.3	0.0 ± 0.0	t = 11.62 (df = 28)	<0.001
	3 months	0.0 ± 0.0	0.0 ± 0.0	Not applicable	Not applicable

Range of motion was analysed separately for extension and flexion. In the immediate postoperative period, an extension lag was observed, averaging 10.0° in the C-TKA group and 8.0° in the RA-TKA group (independent-samples Student's t-test: t = 3.42, df = 28, p = 0.003, significant after Bonferroni correction); this lag resolved completely by the two-week assessment in every patient in both groups and was maintained at all subsequent visits. Active flexion improved progressively in both groups, rising from a mean of 90.0° (C-TKA) and 95.7° (RA-TKA) in the immediate postoperative period to 134.1° and 137.2° respectively at three months. The RA-TKA group showed significantly greater flexion at two weeks, one month, and two months (independent-samples Student's t-test: t = 3.94, 3.94, and 3.95, respectively; df = 28, p < 0.001 at each, all significant after Bonferroni correction). The immediate postoperative difference in flexion (t = 2.21, df = 28, p = 0.042) did not survive correction for the five time-point comparisons and is therefore not interpreted as significant, and by 3 months the between-group difference was not significant (t = 1.83, df = 28, p = 0.057). Range-of-motion data are summarised in Table 4.

**Table 4:** Knee range of motion (extension lag and active flexion) at all follow-up time points. Values are mean ± standard deviation in degrees. An extension lag of 0° denotes full active extension. Between-group comparisons were performed using the independent-samples Student's t-test (df = 28); the value of the t statistic is reported alongside each p-value. Because each outcome was compared at five sequential time points, significance is declared against a Bonferroni-adjusted threshold of p < 0.010; the immediate postoperative flexion difference (p = 0.042) does not meet this threshold and is not interpreted as significant. ROM, range of motion; C-TKA, conventional total knee arthroplasty; RA-TKA, robotic-assisted total knee arthroplasty.

ROM Component	Time Point	C-TKA (mean ± SD)	RA-TKA (mean ± SD)	Test statistic	p-value
Extension lag (degrees)	Immediate post-op	10.0 ± 1.6	8.0 ± 1.6	t = 3.42 (df = 28)	0.003
	2 weeks	0.0 ± 0.0	0.0 ± 0.0	Not applicable	Not applicable
	1 month	0.0 ± 0.0	0.0 ± 0.0	Not applicable	Not applicable
	2 months	0.0 ± 0.0	0.0 ± 0.0	Not applicable	Not applicable
	3 months	0.0 ± 0.0	0.0 ± 0.0	Not applicable	Not applicable
Flexion (degrees)	Immediate post-op	90.0 ± 6.8	95.7 ± 7.3	t = 2.21 (df = 28)	0.042
	2 weeks	100.1 ± 7.0	110.6 ± 7.6	t = 3.94 (df = 28)	<0.001
	1 month	115.1 ± 7.0	125.6 ± 7.6	t = 3.94 (df = 28)	<0.001
	2 months	125.1 ± 7.0	134.0 ± 5.2	t = 3.95 (df = 28)	<0.001
	3 months	134.1 ± 5.6	137.2 ± 3.4	t = 1.83 (df = 28)	0.057

Complications observed during follow-up are summarised in Table 5. The C-TKA group had one superficial surgical-site infection (n = 1/15; 6.7%) and three patients with prolonged pain persisting beyond one month postoperatively (n = 3/15; 20.0%), giving a total complication rate of 26.7% (n = 4/15). The RA-TKA group had one pin-tract infection (n = 1/15; 6.7%) that resolved with local wound care and oral antibiotics; no other infective, thrombotic or prolonged-pain morbidity was observed in this group. All complications recorded in both arms were minor, were managed conservatively, and had completely resolved by the time of the final follow-up. Although the overall complication rate was numerically lower in the RA-TKA arm (n = 1/15; 6.7% versus n = 4/15; 26.7%), this difference did not reach statistical significance on the Fisher exact test (two-tailed p = 0.330; the Fisher exact test does not generate a test statistic). With 15 patients per group, the study had very low power to detect a difference in complication rates, so this result indicates absence of evidence rather than evidence of equivalence.

**Table 5:** Complications observed during the three-month follow-up period in the C-TKA and RA-TKA groups. Data are presented as n (%); percentages are calculated on a denominator of 15 patients in each group. Between-group comparisons were performed using the Fisher exact test (two-tailed), which was chosen because expected cell counts were less than five; the Fisher exact test does not generate a test statistic; therefore, only the exact p-value is reported. C-TKA, conventional total knee arthroplasty; RA-TKA, robotic-assisted total knee arthroplasty.

Complication	C-TKA (n=15)	RA-TKA (n=15)	p-value (Fisher exact test)
Superficial surgical-site infection	1 (6.7%)	0 (0.0%)	1.000
Prolonged pain (>1 month)	3 (20.0%)	0 (0.0%)	0.224
Pin-tract infection	0 (0.0%)	1 (6.7%)	1.000
Deep infection	0 (0.0%)	0 (0.0%)	Not applicable
Total complications	4 (26.7%)	1 (6.7%)	0.330
Complications resolved at last follow-up	4 of 4 (100.0%)	1 of 1 (100.0%)	Not applicable

## Discussion

Conventional total knee arthroplasty relies on manual alignment guides and surgeon-dependent bone preparation, whereas robotic-assisted total knee arthroplasty combines patient-specific preoperative planning with intraoperative robotic guidance [21]. In this prospective non-randomised pilot comparison in an Indian tertiary-care centre, the RA-TKA group had a significantly longer tourniquet time but lower postoperative drain collection, lower early pain scores, higher early Knee Society Scores and greater early flexion than the C-TKA group, with all functional measures converging by 3 months. Three constraints govern every comparison discussed below, and none of them is incidental. The sample was small and not powered for any endpoint; allocation was by patient choice, so the groups differ systematically in affordability and therefore in socio-economic position; and the two groups differed not only in the use of a robotic platform but also in alignment philosophy, mechanical in the C-TKA group and kinematic in the RA-TKA group. The differences observed are accordingly preliminary estimates attributable to a combined treatment strategy, not to robotic assistance, and the discussion that follows is offered as interpretation of pilot data rather than as evidence bearing on current clinical choice.

Alignment philosophy and the limits of attribution

Accurate component positioning and restoration of limb alignment are central objectives of total knee arthroplasty and are associated with implant function and survivorship [22], and published randomised trials and meta-analyses report greater alignment accuracy and reproducibility with robotic assistance than with conventional instrumentation [23-25]. The present study generated no alignment data of its own: postoperative radiographs were not analysed for the hip-knee-ankle axis or for component orientation, and no measure of soft-tissue trauma was recorded. The earlier functional recovery seen in the RA-TKA group therefore cannot be shown to have arisen from greater cutting accuracy, and the evidence cited above describes what robotic platforms have achieved elsewhere rather than what was demonstrated here. Any mechanistic explanation of the present findings, whether based on precision of bone preparation, soft-tissue handling, or the kinematic alignment strategy used in the RA-TKA group, remains untested in this dataset.

Tourniquet time and surgical efficiency

Robotic-assisted TKA requires tracker pin placement, anatomical registration and intraoperative planning, and the RA-TKA group accordingly needed a mean of 17.1 minutes more tourniquet time. This did not correspond to any measured detriment in the outcomes recorded here, although the study was not designed to detect harm related to tourniquet time. The operative-time penalty is known to fall once a team passes the initial learning curve [26], which matters in the Indian context, where theatre throughput in high-volume arthroplasty units is itself a constraint on adoption: a unit performing several TKAs per list absorbs an additional 17 minutes per case differently from one performing two.

Blood loss management

Because a tourniquet was used throughout in both groups, intraoperative blood loss was negligible, and postoperative drain collection was the principal index of perioperative bleeding. Drain output was significantly lower in the RA-TKA group. The mechanism was not investigated: no measure of periarticular tissue disruption, muscle-damage marker or inflammatory index was obtained, and the kinematic alignment strategy used in that group typically requires fewer ligament releases than mechanical alignment, which is an equally plausible explanation. Published work reporting a reduced systemic inflammatory response after robotic-assisted TKA [27] is consistent with the present observation but cannot establish its cause here.

Pain scores and early functional recovery

The RA-TKA group had significantly lower VAS pain scores and higher Knee Society Scores through the early postoperative period, in keeping with previous reports [28,29]. The reason cannot be determined from these data, because the RA-TKA group differed from the C-TKA group in both platform and alignment philosophy and because neither surgical accuracy nor soft-tissue trauma was measured. Functional outcomes in the two groups became comparable by three months, so whatever produced the early advantage did not translate into a medium-term difference; the same convergence has been reported internationally [30]. For the Indian cultural context, this convergence carries particular weight, because the functional endpoints that matter most locally, deep flexion for squatting and cross-legged sitting, were not assessed within the observation window in either group and remain undetermined by this study.

Complications and safety profile

Both surgical techniques demonstrated favourable short-term safety profiles, with only minor complications observed during follow-up. The C-TKA group experienced one superficial surgical-site infection and three cases of prolonged postoperative pain, whereas the RA-TKA group had a single pin-tract infection that resolved with conservative management. Although the overall complication rate was numerically lower in the RA-TKA group, this difference did not reach statistical significance, most likely because of the limited sample size. Pin-site complications remain a recognised concern unique to robotic-assisted procedures; however, published evidence indicates that such complications are uncommon and can generally be prevented through meticulous surgical technique and appropriate postoperative pin-site care [31]. The present data are compatible with short-term safety of both techniques but cannot support any comparative safety claim, because the study was not powered for complication endpoints; adequately sized studies are required before the two techniques can be ranked on safety.

Cost and adoption of RA-TKA in India

Adoption of RA-TKA in India is increasing but remains concentrated in metropolitan and high-volume private institutions, largely because of the capital cost of imported robotic systems [15,16]. In this pilot study, the cost differential was the mechanism by which patients self-selected between the two groups, and it is therefore simultaneously the principal barrier to access and the principal source of confounding in this dataset: the patients who could afford RA-TKA were, by construction, of higher socio-economic status than those who could not, with all the nutritional, occupational and rehabilitation-access differences that this implies. No economic data whatsoever were captured. Itemised hospital bills, implant and consumable costs, robot-specific imaging and disposables, and indirect costs such as time to return to work were not recorded, and no incremental cost-effectiveness ratio was calculated. Every statement about cost in this manuscript is therefore contextual background and carries no quantitative content; no claim of economic advantage or disadvantage is made or implied for either technique. A formal economic evaluation conducted prospectively alongside a randomised comparison is a necessary component of any future trial, and until that is done, the value question in the Indian setting remains open.

Implications for future research

This pilot study yields no clinical recommendation, and none should be drawn from it. What it provides is a set of preliminary estimates usable in designing the trial that would answer the question properly. The between-group differences observed in drain collection, early pain scores and early Knee Society Scores, together with their variances, give the effect sizes from which a formal sample-size calculation can be made; the observed convergence by three months indicates that a meaningful trial must extend well beyond that point; and the complete absence of radiographic and economic data in the present work identifies the two endpoint domains such a trial must add. A future study should be randomised, multi-centre, powered on a single prespecified primary endpoint, analysed with a longitudinal repeated-measures model, and should either hold the alignment philosophy constant across arms or factorialise it so that platform and alignment can be separated. Follow-up of at least one year is the minimum, with the deep flexion, squatting and cross-legged sitting that matter to Indian patients specified as discrete endpoints, and a prospective health-economic evaluation running alongside.

Limitations

This is a pilot study and its limitations are accordingly fundamental rather than peripheral. First, the total sample of 30 patients is underpowered for every endpoint examined; no formal sample-size calculation was performed, and the complication comparison in particular had insufficient power to detect anything but a very large difference, so its non-significant result carries no evidential weight. Second, allocation was not randomised: patients chose their procedure largely on the basis of affordability, which introduces socio-economic selection bias and makes the two groups non-exchangeable at baseline. Third, the intervention was not isolated, since the C-TKA group underwent mechanical alignment and the RA-TKA group underwent kinematic alignment, so every between-group difference reported here reflects the combined effect of platform and alignment philosophy and the design cannot apportion the effect between them. Fourth, no postoperative radiographic alignment measurements were made, so the study contains no direct evidence of surgical accuracy and no claim about precision can be supported from these data. Fifth, no cost or health-economic data were collected, so the economic comparison is entirely unquantified. Sixth, each outcome was compared at five sequential time points; a Bonferroni correction was applied to control the family-wise error rate, but between-group testing at each time point does not account for within-patient correlation, and a longitudinal repeated-measures model would be the more robust approach in a larger dataset. Taken together, these constraints mean that the findings are exploratory and hypothesis-generating, and that no inference about the relative clinical merit of the two techniques can be drawn from them.

Follow-up was limited to three months, which is short for an arthroplasty study given that modern implants are expected to survive 15 years or more. Early differences that have disappeared by three months carry limited clinical weight in the absence of longer-term data on implant survival, late complications and sustained function, and in particular on the deep flexion, squatting and cross-legged sitting that matter in the Indian context and that were not assessed as discrete endpoints here. A minimum of one year of follow-up would be required to comment on sustained functional gain, return to culturally important activities, early wear or loosening, and late complications. Larger multi-centre randomised controlled trials with follow-up of 10-15 years, incorporating radiographic alignment endpoints and a formal economic evaluation, are needed to characterise the relative merits of RA-TKA and C-TKA in the Indian population.

## Conclusions

This was a small, non-randomised pilot study with three months of follow-up. Within these limits, the robotic-assisted group showed a significantly longer tourniquet time, lower postoperative drain collection and pain scores, higher Knee Society Scores at two weeks and one month, and greater early flexion than the conventional group. Functional outcomes converged by three months, and the difference in complication rates was not statistically significant in a sample of this size.

No clinical recommendation follows from a pilot of this size. Conventional TKA remains the reliable, well-validated standard. Given the single-centre, non-randomised design, the confounding of surgical platform with alignment philosophy, and the absence of radiographic and economic data, an adequately powered, randomised, multi-centre trial is required, incorporating radiographic alignment endpoints, a prospective health-economic evaluation and discrete outcomes relevant to the local population, to determine whether robotic assistance has a place in Indian arthroplasty practice.
